# Adipocytokines, Hepatic and Inflammatory Biomarkers and Incidence of Type 2 Diabetes. The CoLaus Study

**DOI:** 10.1371/journal.pone.0051768

**Published:** 2012-12-12

**Authors:** Pedro Marques-Vidal, Rémy Schmid, Murielle Bochud, François Bastardot, Roland von Känel, Fred Paccaud, Jennifer Glaus, Martin Preisig, Gérard Waeber, Peter Vollenweider

**Affiliations:** 1 Institute of Social and Preventive Medicine (IUMSP), Lausanne University Hospital, Lausanne, Switzerland; 2 Department of Medicine, Internal Medicine, CHUV and Faculty of Biology and Medicine, Lausanne, Switzerland; 3 Division of Psychosomatic Medicine, Bern University Hospital, Inselspital, and University of Bern, Bern, Switzerland; 4 Department of Psychiatry, CHUV, Lausanne, Switzerland; German Diabetes Center, Leibniz Center for Diabetes Research at Heinrich Heine University, Germany

## Abstract

**Context:**

There is contradictory information regarding the prognostic importance of adipocytokines, hepatic and inflammatory biomarkers on the incidence of type 2 diabetes. The objective was to assess the prognostic relevance of adipocytokine and inflammatory markers (C-reactive protein – CRP; interleukin-1beta – IL-1β; interleukin-6– IL-6; tumour necrosis factor-α – TNF-α; leptin and adiponectin) and gamma-glutamyl transpeptidase (γGT) on the incidence of type 2 diabetes.

**Methods:**

Prospective, population-based study including 3,842 non-diabetic participants (43.3% men, age range 35 to 75 years), followed for an average of 5.5 years (2003–2008). The endpoint was the occurrence of type 2 diabetes.

**Results:**

208 participants (5.4%, 66 women) developed type 2 diabetes during follow-up. On univariate analysis, participants who developed type 2 diabetes had significantly higher baseline levels of IL-6, CRP, leptin and γGT, and lower levels of adiponectin than participants who remained free of type 2 diabetes. After adjusting for a validated type 2 diabetes risk score, only the associations with adiponectin: Odds Ratio and (95% confidence interval): 0.97 (0.64–1.47), 0.84 (0.55–1.30) and 0.64 (0.40–1.03) for the second, third and forth gender-specific quartiles respectively, remained significant (P-value for trend = 0.05). Adding each marker to a validated type 2 diabetes risk score (including age, family history of type 2 diabetes, height, waist circumference, resting heart rate, presence of hypertension, HDL cholesterol, triglycerides, fasting glucose and serum uric acid) did not improve the area under the ROC or the net reclassification index; similar findings were obtained when the markers were combined, when the markers were used as continuous (log-transformed) variables or when gender-specific quartiles were used.

**Conclusion:**

Decreased adiponectin levels are associated with an increased risk for incident type 2 diabetes, but they seem to add little information regarding the risk of developing type 2 diabetes to a validated risk score.

## Introduction

The prevalence of type 2 diabetes is increasing worldwide [1]. Interleukin-1 beta (IL-1β), interleukin 6 (IL-6), and C-reactive protein (CRP) are three inflammatory markers which have been shown to predict the development of diabetes [2], [3], [4], although this association is interpreted controversially [5], [6]. Conversely, tumour necrosis factor alpha (TNF-α) has not been reported to affect diabetes development [3]. Adipocytokines also contribute to the development of type 2 diabetes; for instance, leptin increases [7], while adiponectin decreases [8] the risk of type 2 diabetes. Finally, recent studies have suggested that gamma-glutamyl transpeptidase (γGT) might be a better predictor of type 2 diabetes than inflammatory markers [9], [10], [11]. Still, although most studies included age, gender and anthropometric data (body mass index or waist circumference), other variables such as alcohol consumption or family history of diabetes were not considered. Recently, several type 2 diabetes risk scores have been proposed, based on clinical data alone, or on clinical variables in combination with biological parameters [12], [13].

In a previous study, we have demonstrated that the Kahn (clinical + biological) risk score [14] adequately predicted the type 2 diabetes incidence within the CoLaus cohort [13]. The aim of this prospective observational study was twofold: 1) to assess the associations of various adipocytokines, hepatic and inflammatory markers with the incidence of type 2 diabetes; and 2) to assess the value of adding these markers into an existing type 2 diabetes risk score to possibly improve the prediction of type 2 diabetes, as recommended by current guidelines [15], [16]. To our knowledge, there is little information about whether adipocytokines, hepatic and inflammatory markers might improve existing type 2 diabetes risk scores.

## Methods

### Recruitment

The CoLaus Study was designed to assess the prevalence of cardiovascular risk factors (hypertension, diabetes, dyslipidemia, obesity and smoking) and to identify new molecular determinants of these risk factors in the Caucasian population from Lausanne (Switzerland). The study was approved by the Institutional Ethics Committee of the University of Lausanne and all participants provided written informed consent into the protocol.

The sampling procedure of the CoLaus Study has been described previously [17]. In summary, a simple, nonstratified random sample of the overall population of Lausanne was drawn. The following inclusion criteria were applied: (a) written informed consent; (b) willingness to take part in the examination and to provide blood samples and (c) Caucasian origin. Recruitment began in June 2003 and ended in May 2006. Participation rate was 41% with 6,188 Caucasian participants (3,251 women and 2,937 men) taking part in the genetic study.

All participants were seen in the morning after an overnight fast (minimum fasting time 8 hours).

### Baseline data collection

The participants were asked about their personal and family history of diabetes and hypertension as well as their treatment.

Body weight and height were measured with participants standing without shoes in light indoor clothes. Body weight was measured in kilograms to the nearest 100 g using a Seca® scale, which was calibrated regularly. Height was measured to the nearest 5 mm using a Seca® height gauge. Waist circumference was measured with a non-stretchable tape over the unclothed abdomen at the narrowest point between the lowest rib and the iliac crest. Two measures were made and the mean (expressed in centimetres) was used for analyses. Blood pressure and resting pulse were measured thrice on the left arm, with an appropriately sized cuff, after a rest of at least 10 minutes in the seated position using an Omron® HEM-907 automated oscillometric sphygmomanometer. The average of the last two blood pressure measurements was used for analyses. Hypertension was defined as systolic blood pressure ≥140 mm Hg and/or diastolic blood pressure ≥90 mm Hg or presence of antihypertensive drug treatment.

Venous blood samples (50 mL) were drawn in the fasting state. Serum was preferred to plasma as it has been shown that different anticoagulants may affect absolute cytokine levels differently [18]. It has also been stated that cytokine levels in a single blood sample may be useful biomarkers of inflammation in population-based studies of obesity-related diseases [18]. Serum samples were kept at −80°C for five years before assessment of the other cytokines and sent on dry ice to the laboratory. Adiponectin was assessed by ELISA (R&D Systems, Inc, Minneapolis, USA) with a maximum inter-assay CV of 8.3% and a maximum intra-assay CV of 8.3%. Leptin was assessed by ELISA (American Laboratory Products Company, Windham, USA) with a maximum inter-assay CV of 12.8% and a maximum intra-assay CV of 5.8%; glucose by glucose dehydrogenase (2.1%–1.0%); γGT by the optimized standard method according to the International Federation of Clinical Chemistry, at 37°C (1.6%–0.4%); HDL-cholesterol by CHOD-PAP + PEG + cyclodextrin (3.6%–0.9%); triglycerides by GPO-PAP (2.9%–1.5%) and uric acid by uricase-PAP (1.0%–0.5%).

High sensitive CRP (hs-CRP) was assessed by immunoassay and latex HS (IMMULITE 1000–High, Diagnostic Products Corporation, LA, CA, USA) with maximum intra- and interbatch coefficients of variation of 1.3% and 4.6%, respectively. For cytokine measurement, serum was preferred to plasma as it has been shown that different anticoagulants may affect absolute cytokine levels differentially [19]. Serum samples were kept at −80°C before assessment of the other cytokines and sent on dry ice to the laboratory. Cytokine levels were measured using a multiplexed particle-based flow cytometric cytokine assay [20], a methodology also used in other studies [21]. Milliplex kits were purchased from Millipore (Zug, Switzerland). The procedures closely followed the manufacturer's instructions. The analysis was conducted using a conventional flow cytometer (FC500 MPL, BeckmanCoulter, Nyon, Switzerland). Lower detection limits for IL-1β, IL-6 and TNF-α were 0.2 ng/l. A good agreement between signal and cytokine was found within the assay range (R^2^≥0.99). Intra and inter-assay coefficients of variation were 15% and 16.7% for IL-1β, 16.9% and 16.1% for IL-6 and 12.5% and 13.5% for TNF-α, respectively. Repeated measurements were conducted in 80 subjects randomly drawn from the initial sample; Spearman rank correlations between duplicate measurements were 0.91, 0.96 and 0.89 for IL-1β, IL-6 and TNF-α (all p<0.001).

### Follow-up data collection

In their original consent letter, participants were informed of a potential follow-up study. Approximately 96% gave permission and were re-contacted. Prior to the follow-up interview, participants received the detailed information letter and consent forms.

The follow-up visit was performed five years after the collection of baseline data. Similarly to the baseline evaluation, the somatic investigation included an interview, a physical exam, blood analysis and a set of questionnaires. At the time of the analysis, data for 4,602 participants was available.

In both evaluation periods (baseline and follow-up) diabetes was defined as fasting plasma glucose (FPG) ≥7.0 mmol/L and/or presence of oral hypoglycemic or insulin treatment [22]. Type 2 diabetes was defined in case of diabetes without self-reported type 1 diabetes. Subjects who presented with type 1 or type 2 diabetes at baseline were excluded from the present analysis.

### Statistical analysis

Of the initial 4,602 participants with follow-up data, 269 (5.8%) were excluded because of type 1 or type 2 diabetes at baseline, 85 (1.8%) because of missing fasting glucose data at follow-up and 406 (8.8%) because of missing data for leptin (N = 386) or adiponectin (N = 20), leaving 3842 participants (83.5%) for analysis. Excluded participants were less frequently women, were younger and had lower body mass index and hs-CRP levels than non-excluded ones, while no significant differences were found regarding the other biological markers (**Table S1**).

Statistical analysis was conducted using Stata v.12.1 (Stata Corp, College Station, TX, USA). Biomarkers were presented as median and (interquartile range) of measured values, percentage of values below LOD and percentage of values within each gender-specific quartile. Many participants had cytokine levels below LOD (37% for IL-1β, 8.8% for IL-6 and 1.6% for TNF-α). Undetectable values were included in the first quartile. For participants with undetectable cytokines levels, a value of half the LOD was assigned for quantitative analyses [23], [24]. Comparisons between subjects who developed type 2 diabetes and those who did not were conducted using Kruskall-Wallis non-parametric test or chi-square. Association with incident diabetes was analyzed by logistic regression and the results were expressed for each variable as odds ratio with 95% confidence interval. Three different models were tested: 1) adjusting for age and gender; 2) adjusting for age, gender and body mass index (BMI); and 3) adjusting for a type 2 diabetes risk score [14] validated in the CoLaus cohort [25]. It includes age, family history of type 2 diabetes, black race (not used here as all participants are Caucasian), height, waist circumference, resting heart rate, presence of hypertension, HDL cholesterol, triglycerides, fasting glucose and serum uric acid. Age, gender and BMI were used as covariates because they are associated with an increased risk for type 2 diabetes. The main aim of this study was to assess the predictive utility of several biomarkers regarding the development of type 2 diabetes relative to a validated risk score for type 2 diabetes.

As measures of overall model fit, we examined the Hosmer-Lemeshow goodness-of-fit test, the Akaike Information Criterion (AIC) [26] and the Bayes Information Criterion (BIC) [27]; the lower the AIC and BIC values, the better the goodness of fit. The improvement in the predictive ability of this risk score by inclusion of adipocytokine, hepatic or inflammatory data was assessed by comparing the areas under the receiver operating curve (AROC) between the models with and without the adipocytokine, hepatic or inflammatory marker of interest. Two AROCs were computed: 1) using the type 2 diabetes risk predicted by the model as a continuous variable and 2) splitting the type 2 diabetes risk into two categories (not at risk and at risk). For this second AROC, a threshold of 23% was used for sensitivity analyses. This was decided as the original 46% threshold had been proposed for a 10-year follow-up [14], while the average follow-up in this study was only 5 years. We also assessed the sensitivity, specificity, positive and negative predictive values, net reclassification improvement (NRI) and integrated discrimination improvement (IDI) [28] (with 95% confidence intervals) for each adipocytokine, hepatic or inflammatory marker (divided into sex-specific quartiles), using incident diabetes and the binary variable defined previously, i.e. “not at risk” (estimated risk <23%) and “at risk” (estimated risk≥23%). The AUC, NRI and IDI are presented for each adipocytokine, hepatic or inflammatory marker as recommended [29]. Conversely, we did not present continuous NRI as it might not be and adequate marker of model improvement [30].

Sensitivity analyses were conducted as follows: analyses were conducted using continuous, log-transformed values instead of quartiles, and assessing the OR for the increase in one standard deviation of the log-transformed values. As leptin, adiponectin and γGT showed considerable variation between men and women, we also tested the use of sex-specific quartiles for these variables. As it has been suggested that the simultaneous inclusion of several markers might improve risk prediction [31], [32], [33], the improvement in predictive ability was also assessed for the following combinations: adiponectin and CRP, CRP and γGT, and all inflammatory markers. Finally, a prospective study suggested that IL-1β might interact with IL-6, participants with detectable IL-1β levels and elevated IL-6 levels presenting an increased risk to develop type 2 diabetes relative to individuals with undetectable IL-1β levels and increased IL-6 levels [3], a similar analysis was performed.

Statistical significance was established for p<0.05.

## Results

### Association between cytokines and incident type 2 diabetes mellitus

During the 5-year follow-up, 208 of the 3842 participants (5.4%, 95% confidence interval 4.7–6.2) developed type 2 diabetes, corresponding to incidence rate of 9.8 cases per 1,000 persons-year. The levels of adipocytokines, hepatic and inflammatory markers in the group who developed type 2 diabetes and in the group who did not are summarized in **Table 1** **and Table S2**. Participants who developed type 2 diabetes during follow-up had significantly higher levels of IL-6, hs-CRP, leptin and γGT, and lower levels of adiponectin than participants who remained free from type 2 diabetes. No significant differences were found for TNF-α and IL-1β.

**Table 1:** Baseline levels of adipocytokine, hepatic and inflammatory markers for participants who developed type 2 diabetes (n = 208) and those who remained free from it (n = 3634) after 5 years of follow-up.

	Non diabetic	Incident diabetes	P-value
Age (years)	52.1±10.5	57.1±9.5	<0.001
Women (%)	58.1	31.7	<0.001
Body mass index (kg/m^2^)	25.2±3.9	29.3±4.2	<0.001
Interleukin 1β quartiles			
First	37.0	43.8	
Second	21.3	22.1	0.11
Third	20.9	19.2	
Fourth	20.8	14.9	
Interleukin 6 quartiles			
First	26.0	16.8	
Second	25.7	27.9	0.03
Third	24.6	28.4	
Fourth	23.6	26.9	
Tumour necrosis factor-α quartiles			
First	26.0	21.6	
Second	25.4	26.4	0.48
Third	24.4	27.9	
Fourth	24.2	24.0	
hs-CRP quartiles			
First	29.2	10.6	
Second	25.5	18.7	<0.001
Third	24.3	30.9	
Fourth	21.0	40.4	
Leptin quartiles			
First	27.5	8.7	
Second	26.4	21.6	<0.001
Third	24.7	22.6	
Fourth	21.4	47.1	
Adiponectin quartiles			
First	23.2	34.1	
Second	25.1	27.9	<0.001
Third	25.3	23.1	
Fourth	26.4	14.9	
γGT quartiles			
First	29.6	10.6	
Second	25.1	17.8	<0.001
Third	24.8	30.8	
Fourth	20.5	40.8	

LOD, lower limit of detection; CRP, C-reactive protein; γGT, gamma-glutamyl transpeptidase. Results are expressed as % of column total. Statistical analysis by Student's t-test chi-square or Fisher's exact test.

The association between adipocytokine, hepatic and inflammatory marker levels and incident type 2 diabetes was further assessed by analysing the risk of developing type 2 diabetes according to the quartiles of each biological variable. The results are summarized in **Table 2**. On bivariate analysis, IL-6, hs-CRP, leptin and γGT were positively associated with increased risk of type 2 diabetes; IL-1β and adiponectin were inversely associated with type 2 diabetes risk. Conversely, no significant association was found with TNF-α. After multivariate adjustment, only the positive association with γGT and the negative association with adiponectin retained their significance (**Table 2**).

**Table 2:** Association between adipocytokine, hepatic or inflammatory marker levels and incident type 2 diabetes.

	Adjusted ^a^	P-value	Adjusted ^b^	P-value	Adjusted ^c^	P-value
Quartiles of IL-1β						
First	1 (ref)		1 (ref)		1 (ref)	
Second	0.95 (0.66–1.38)		0.95 (0.65–1.40)		0.95 (0.62–1.44)	
Third	0.86 (0.58–1.27)	0.08	0.79 (0.53–1.18)	0.12	0.72 (0.47–1.10)	0.12
Fourth	0.69 (0.45–1.06)		0.74 (0.48–1.14)		0.74 (0.47–1.18)	
Quartiles of IL-6						
First	1 (ref)		1 (ref)		1 (ref)	
Second	1.54 (1.00–2.39)		1.29 (0.82–2.01)		1.44 (0.89–2.33)	
Third	1.58 (1.02–2.45)	0.05	1.22 (0.78–1.92)	0.52	1.14 (0.71–1.84)	0.72
Fourth	1.58 (1.02–2.45)		1.19 (0.75–1.88)		1.18 (0.73–1.93)	
Quartiles of TNF-α						
First	1 (ref)		1 (ref)		1 (ref)	
Second	1.24 (0.82–1.86)		1.21 (0.80–1.85)		1.01 (0.64–1.59)	
Third	1.23 (0.82–1.84)	0.87	1.07 (0.70–1.63)	0.39	0.85 (0.54–1.34)	0.054
Fourth	1.04 (0.68–1.59)		0.85 (0.55–1.32)		0.65 (0.41–1.04)	
Quartiles of hs-CRP						
First	1 (ref)		1 (ref)		1 (ref)	
Second	1.91 (1.12–3.26)		1.55 (0.90–2.67)		1.25 (0.70–2.21)	
Third	2.88 (1.74–4.75)	<0.001	1.87 (1.12–3.13)	<0.001	1.31 (0.76–2.25)	0.12
Fourth	4.63 (2.85–7.53)		2.35 (1.41–3.93)		1.53 (0.90–2.60)	
Quartiles of leptin						
First	1 (ref)		1 (ref)		1 (ref)	
Second	2.50 (1.43–4.36)		1.91 (1.08–3.35)		1.63 (0.90–2.94)	
Third	2.65 (1.52–4.62)	<0.001	1.54 (0.87–2.73)	0.02	0.93 (0.51–1.69)	0.77
Fourth	6.35 (3.79–10.7)		2.23 (1.25–3.98)		1.32 (0.75–2.32)	
Quartiles of adiponectin						
First	1 (ref)		1 (ref)		1 (ref)	
Second	0.68 (0.47–0.98)		0.74 (0.50–1.08)		0.97 (0.64–1.47)	
Third	0.54 (0.37–0.80)	<0.001	0.62 (0.41–0.92)	<0.001	0.84 (0.55–1.30)	0.05
Fourth	0.30 (0.19–0.47)		0.41 (0.26–0.65)		0.64 (0.40–1.03)	
Quartiles of γGT						
First	1 (ref)		1 (ref)		1 (ref)	
Second	1.78 (1.04–3.06)		1.39 (0.80–2.41)		1.07 (0.60–1.91)	
Third	2.78 (1.69–4.59)	<0.001	1.79 (1.07–3.00)	<0.001	1.13 (0.66–1.94)	0.17
Fourth	4.56 (2.80–7.42)		2.88 (1.75–4.75)		1.45 (0.85–2.46)	

IL-1β, interleukin 1-β; IL-6, interleukin-6; TNF-α, tumour necrosis factor-α; CRP, C-reactive protein; γGT, gamma-glutamyl transpeptidase. Data from 208 participants who developed type 2 diabetes mellitus and 3634 controls. Results are expressed as Odds ratio and (95% confidence interval). Statistical analysis by logistic regression: ^a^, adjusting for age and gender; ^b^, adjusting for age, gender and body mass index; ^c^, adjusting for Kahn's clinical and biological score (including age, family history of type 2 diabetes, height, waist circumference, resting heart rate, presence of hypertension, HDL cholesterol, triglycerides, fasting glucose and serum uric acid).

### Impact in predictive capacity of a type 2 diabetes mellitus risk score

The improvement in the predictive ability of this risk score by inclusion of adipocytokine, hepatic or inflammatory data is summarized in **Tables 3** **and** **4**. Overall, all models fitted to the data, as indicated by the nonsignificant results of the Hosmer-Lemeshow test. Conversely, none of the markers led to an improvement in goodness of fit as indicated by the AIC and BIC. Also, no improvement in the predictive capacity of the initial model, as assessed by AROC, was found using type 2 diabetes risk either as a continuous or a “not at risk” (<23%) and “at risk” (≥23%) category (**Table 3**). No significant improvement was found in sensitivity, specificity, NPV, PPV and NRI for all the markers studied (**Table 4**), although the improvement in IDI for adiponectin was close to statistical significance (p = 0.052). Conversely, including all markers in the model led to an improvement in the AROC (**Table 3**) and in IDI but not in NRI (**Table 4**).

**Table 3:** Impact of adding different adipocytokine, hepatic or inflammatory markers as quartiles in the predictive capacity of a clinical + biological (C+B) risk score for type 2 diabetes.

	HL-test (p-value)	AIC	BIC	AROC § (95% CI)	AROC §§ (95% CI)
Kahn's C+B score	0.84	1123.6	1136.1	0.901 (0.883–0.919)	0.681 (0.648–0.715)
Kahn's C+B score + IL-1β	0.97	1126.3	1157.6	0.902 (0.884–0.920)	0.692 (0.658–0.726)
Kahn's C+B score + IL-6	0.96	1127.1	1158.4	0.901 (0.883–0.919)	0.684 (0.650–0.717)
Kahn's C+B score + TNF-α	0.95	1124.9	1156.2	0.902 (0.883–0.920)	0.686 (0.652–0.720)
Kahn's C+B score + hs-CRP	0.47	1126.9	1158.2	0.901 (0.883–0.919)	0.686 (0.653–0.720)
Kahn's C+B score + leptin	0.99	1122.9	1154.2	0.901 (0.883–0.919)	0.682 (0.648–0.715)
Kahn's C+B score + adiponectin	0.56	1125.4	1156.7	0.902 (0.884–0.920)	0.688 (0.654–0.722)
Kahn's C+B score + γGT	0.78	1126.5	1157.8	0.902 (0.884–0.920)	0.688 (0.654–0.722)
Kahn's C+B score + all variables	1.00	1137.9	1281.8	0.908 (0.891–0.925) **	0.699 (0.665–0.733)

Statistical analysis by logistic regression. Each line shows the results of the original model (first line with HL, Hosmer-Lemeshow goodness-of-fit test (only p-values are reported); AIC, Akaike's information criterion; BIC, Bayesian information criterion; AROC, area under the ROC curve; IL-1β, interleukin 1 beta; IL-6, interleukin 6; TNF-α, tumour necrosis factor alpha; hs-CRP, high sensitive C reactive protein; γGT, gamma glutamyl transpeptidase. **§** using the type 2 diabetes risk predicted by the model as a continuous variable; **§§** splitting the type 2 diabetes risk into two categories (not at risk and at risk). Data from 208 participants who developed type 2 diabetes mellitus and 3634 controls. ** significantly different (p<0.01) from the baseline model (Kahn's C+B score).

**Table 4:** Impact of adding different adipocytokine, hepatic or inflammatory markers as quartiles in the ability of a clinical + biological risk score to predict type 2 diabetes, using a 23% probability threshold to define high risk subjects.

	Sensitivity (%)	Specificity (%)	PPV (%)	NPV (%)	NRI (%)	IDI (%)
Kahn's C+B score	40.9 (34.1–47.9)	95.4 (94.6–96.0)	33.6 (27.8–39.8)	96.6 (95.9–97.1)	-	-
Score + IL-1β	42.8 (36.0–49.8)	95.7 (94.9–96.3)	36.0 (30.0–42.4)	96.7 (96.1–97.3)	2.20 (−0.13; 4.53)	0.22 (−0.10; 0.55)
Score + IL-6	41.3 (34.6–48.4)	95.4 (94.6–96.0)	33.9 (28.1–40.0)	96.6 (96.0–97.2)	0.48 (−2.66; 3.62)	0.00 (−0.26; 0.27)
Score + TNF-α	41.8 (35.0–48.8)	95.3 (94.6–96.0)	34.0 (28.2–40.1)	96.6 (96.0–97.2)	0.93 (−2.35; 4.22)	0.25 (−0.14; 0.63)
Score + hs-CRP	41.8 (35.0–48.8)	95.5 (94.7–96.1)	34.5 (28.7–40.7)	96.6 (96.0–97.2)	1.04 (−1.27; 3.36)	0.21 (−0.06; 0.48)
Score + leptin	40.9 (34.1–47.9)	95.5 (94.8–96.1)	34.1 (28.3–40.4)	96.6 (95.9–97.1)	0.11 (−2.58; 2.80)	0.36 (−0.05; 0.78)
Score + adiponectin	41.8 (35.0–48.8)	95.7 (95.0–96.4)	36.0 (29.9–42.3)	96.6 (96.0–97.2)	1.67 (−1.96; 4.59)	0.31 (0.00; 0.62)§
Score + γGT	42.3 (35.5–49.3)	95.3 (94.6–96.0)	34.0 (28.2–40.1)	96.7 (96.0–97.2)	1.36 (−1.15; 3.87)	0.17 (−0.15; 0.49)
Score + all variables	44.2 (37.4–51.3)	95.5 (94.8–96.1)	35.9 (30.1–42.1)	96.8 (96.1–97.3)	3.85 (−0.39; 8.09)	2.09 (1.08; 3.10)*

Results are expressed as percentage and (95% confidence interval). PPV, positive predictive value; NPV, negative predictive value; NRI, net reclassification improvement; IDI, integrated discrimination improvement; IL-1β, interleukin 1 beta; IL-6, interleukin 6; TNF-α, tumour necrosis factor alpha; hs-CRP, high sensitive C reactive protein; γGT, gamma glutamyl transpeptidase. Data from 208 participants who developed type 2 diabetes mellitus and 3634 controls. § p-value 0.052; *, p-value<0.01.

### Sensitivity analyses

The results of the analyses using continuous, log-transformed values of the different markers are summarized in **Tables S3, S4, S5**. On bivariate analysis, IL-6, hs-CRP, leptin and γGT were positively associated with increased risk of type 2 diabetes; IL-1β and adiponectin were inversely associated with type 2 diabetes risk. Conversely, no significant association was found with TNF-α. After multivariate adjustment, only the positive association with γGT and the negative association with adiponectin retained their significance (**Table 2**).

After stratification on IL-1β levels, no differences were found regarding the association between IL-6 and incident type 2 diabetes (**not shown**). When sex-specific quartiles for leptin, adiponectin and γGT were used no significant improvement was found in sensitivity, specificity, NPV, PPV and NRI for all the markers studied (**not shown**). Similar findings were observed for most of the different combinations of markers (adiponectin and CRP, CRP and γGT, and all inflammatory markers, **Table S6**), which did not improve any goodness of fit or predictive capacity (CRP + γGT) or improved the IDI but improved neither goodness of fit nor the AUC (CRP and adiponectin). The inclusion of all 7 markers in the model significantly improved the AROC and the IDI, but not the NRI (**Table S6**).

## Discussion

Our results indicate that low adiponectin levels are associated with increased risk for type 2 diabetes after adjusting for a validated type 2 diabetes risk score. Conversely, none of the markers studied improved prediction compared to the type 2 diabetes risk score, suggesting that these markers, although associated with type 2 diabetes, do not add clinically useful information regarding the risk of developing type 2 diabetes.

As a whole, our results suggest that the effect of cytokines (IL-1β, IL-6 and TNF-α) on the incidence of type 2 diabetes might be mediated by other factors; they also suggest that these markers do not improve risk prediction as assessed by a validated type 2 diabetes risk score. A prospective study suggested that IL-1β might interact with IL-6, participants with detectable IL-1β levels and elevated IL-6 levels presenting an increased risk to develop type 2 diabetes relative to individuals with undetectable IL-1β levels and increased IL-6 levels [3]. Indeed, after stratification on IL-1β levels, similar findings were obtained (**not shown**). Participants who developed type 2 diabetes had higher baseline IL-6 levels than participants who did not, but the association was no longer significant after multivariate adjustment, a finding also reported by others [34]. Finally, no association between TNF-α levels and type 2 diabetes was found, a finding in agreement with the literature [3], [35].

CRP is an inflammatory marker that has been associated with increased incidence of type 2 diabetes in several studies [2], [36], prompting the inclusion of hs-CRP in a type 2 diabetes risk prediction score [33]. In this study, a highly significant positive association was found between hs-CRP levels and incident type 2 diabetes, but this association was no longer significant after multivariate adjustment, a finding also reported previously [5], [37]. Again, adding hs-CRP quartiles to the validated risk score did not improve risk prediction, suggesting that the effect of hs-CRP on the development of type 2 diabetes is either mediated by one of the components of the risk score, or that the independent effect of hs-CRP is rather small. Further studies are needed to confirm these findings.

Leptin has been shown to be an independent predictor of type 2 diabetes in some [38], [39] but not in all studies [40], [41]. Still, some studies excluded the middle tertile from the analyses [39] and, most importantly, none of these studies provided information regarding NRI or IDI as recommended [15], [16]. In the present study, leptin was significantly associated with type 2 diabetes risk after adjusting for age, gender and BMI, but this association was no longer significant after adjusting for the type 2 diabetes risk score. Also, no improvement in the area under the ROC, NRI and IDI was noted. It is possible that leptin is only one of the several mediators of obesity or excess adiposity on the incidence of type 2 diabetes [40], so the information for type 2 diabetes risk assessment provided by assessing leptin levels is smaller than by assessing adiposity. Another possible explanation is that the Kahn C+B score includes serum glucose, a strong predictor of risk of diabetes, which was not available in MONICA/KORA [39].

In the present study, we confirmed that high adiponectin levels were associated with a lower risk of developing type 2 diabetes, a finding in agreement with the literature [7], [8], [42], [43]. Conversely, adding adiponectin to a validated type 2 diabetes risk score led to no significant improvement in risk prediction, in agreement with a previous study [44]. Interestingly, in a previous study [43], adiponectin significantly increased C-index and NRI in one cohort (FINRISK97) but not in another (Heart 2000), and only the combination of adiponectin with other markers significantly improved NRI [43]. Although most of these studies improved the knowledge of the physiopathology of type 2 diabetes, it remains to be assessed if the increased costs due to biomarker assessment would be compensated by increased savings due to a better management of patients at high risk for type 2 diabetes. Further, a score including adiponectin and other biomarkers as proposed previously [43] would be difficult to apply in clinical practice, as it would need to assess different biomarkers in men and women (ferritin and IL-1 receptor antagonist for women and CRP and insulin for men). Indeed, in a recent study, about 50% and 70% of German general practitioners explicitly state not to use guidelines or risk calculators, respectively [45]. Hence, future studies should assess not only the importance of new markers in improving the prediction of type 2 diabetes but also their applicability in clinical practice and their cost-effectiveness.

A strong positive association between γGT levels and risk of developing type 2 diabetes was found, and this association persisted after multivariate adjustment. These findings are in agreement with the literature [9], [10], [36], [46], suggesting potential interactions between γGT, enhanced hepatic neoglucogenesis and/or early alterations of insulin secretion [10], [47]. Still, contrary to another study [33], adding γGT quartiles to the type 2 diabetes risk prediction model failed to improve prediction, suggesting that γGT assessment might not be necessary for predicting type 2 diabetes risk in clinical practice. Still, it will be of interest to evaluate the clinical interest of hepatic markers for type 2 diabetes risk prediction, as this assessment might also be used for other purposes, such as the assessment of nonalcoholic-fatty-liver-disease [48].

In this study, the effect of each biological marker was assessed individually, and it has been suggested that the simultaneous inclusion of several markers might improve risk prediction [31], [32], [33]. For instance, a recent study showed that the combined use of adiponectin and CRP led to an increased risk prediction [31]. Still, including these two markers simultaneously in the model did not lead to an improvement in the AROC. Similarly, adding simultaneously CRP and γGT quartiles as suggested in another study [33] also led to a non-significant change in AROC and in the NRI. Another study [43] showed that gender specific scores including adiponectin and apolipoprotein B (for both genders), ferritin and IL-1 receptor antagonist (for women) and CRP and insulin (for men) significantly improved risk prediction but, as indicated previously, the use of a gender-specific risk score might not be easy to implement in clinical practice. Further, as no data was available regarding IL-1 receptor antagonist in our study, we could not replicate these findings. Finally, a study has shown that the simultaneous addition of 13 inflammation-related biomarkers led to a significant improvement in risk prediction compared to a base model including age, sex and survey [32]. Indeed, including all markers (as quartiles) simultaneously in the model led to a significant improvement in the AROC and IDI but failed to improve NRI. Although it can be argued that the risk improvement was borderline significant, it remains to be assessed if the evaluation of several different inflammation-related biomarkers leads to a sufficient improvement in type 2 diabetes risk estimation to be cost-effective and easily implemented in clinical practice.

Our study has several limitations. The participation rate was low (41%), which might limit the generalization of the findings; however, this participation rate is similar to other epidemiological studies [49]. Follow-up time was limited to 5 years; still, a lower threshold regarding the risk of type 2 diabetes was used to account for this lower follow-up period. Hence, a 23% risk threshold was chosen because our follow-up time was only 5 years, which is half the 46% threshold proposed by the original Kahn C+B equation, which was based on a 10 year follow-up. It has been also suggested that the integrated discrimination improvement might not be a valid tool for evaluating the capacity of a marker to predict a binary outcome of interest, because he standard error of the IDI estimate tends to underestimate the error [50]. This statement actually strengthens our conclusions, as the confidence intervals for the non-significant IDI might be even larger than reported. Further, this study was limited to Caucasian participants and whether the results also apply to other ethnicities is currently unknown. It has also been stated that NRI is dependent on cut-off values [51], although this statement has been challenged [52]. It should also be noted that, despite their inclusion on recent guidelines [15], [16], it has been recently shown that NRI and IDI interpretation should be made with caution [29], [30]. Most biomarkers studied presented a relatively large intra- and inter-batch variability, and some also present a large intraindividual variability [53]. This would lead to an increase in the variance of estimated risk, with a consequent decrease in discrimination [54]. This might partly explain the lack of association between some markers and type 2 diabetes after multivariate adjustment, and it is possible that improvements will be observed if more stable markers are used. Also, no OGTT were performed to diagnose type 2 diabetes and no antibodies were determined to exclude type 1 diabetes either at baseline or at follow-up. Finally, including in the analyses the 406 participants without leptin and adiponectin data led to similar conclusions regarding cytokines and γGT (**not shown**).

In summary, our results suggest that elevated baseline adiponectin levels are negatively associated with increased risk for type 2 diabetes. Still, these markers do not significantly improve the prediction capacity of an existing, validated type 2 diabetes risk score. Further research should confirm if our findings also apply to other populations or to other settings. Finally, additional research on new determinants of type 2 diabetes is of importance, as it might lead to new therapeutic pathways and/or to the identification of new prognostic markers of type 2 diabetes.

## Supporting Information

Table S1 **Comparison between participants included and participants excluded because of missing baseline leptin or adiponectin data.**
(DOC)

Table S2 **Cytokine distribution between participants with (N = 208) and without (N = 3634) incident diabetes.**
(DOC)

Table S3 **Association between adipocytokine, hepatic or inflammatory marker levels (as continuous log-transformed variables) and incident type 2 diabetes.**
(DOC)

Table S4 **Impact of adding different adipocytokine, hepatic or inflammatory markers (as continuous log-transformed variables) in the predictive capacity of a clinical + biological (C+B) risk score for type 2 diabetes.**
(DOC)

Table S5 **Impact of adding different adipocytokine, hepatic or inflammatory markers as continuous log-transformed variables in the ability of a clinical + biological risk score to predict type 2 diabetes, using a 23% probability threshold to define high risk subjects.**
(DOC)

Table S6 **Impact of adding specific combinations of adipocytokine, hepatic or inflammatory markers in the predictive capacity of a clinical + biological** (**C+B**) **risk score for type 2 diabetes, using a 23% probability threshold to define high risk subjects.**
(DOC)
