# Supplementary material for: Adipocytokines, Hepatic and Inflammatory Biomarkers and Incidence of Type 2 Diabetes. The CoLaus Study
Source: PLoS One. 2012 Dec 12;7(12):e51768. doi: 10.1371/journal.pone.0051768 (PMC3520903; doi:10.1371/journal.pone.0051768)
Supplement: Table S1 — Comparison between participants included and participants excluded because of missing baseline leptin or adiponectin data. (DOC) [file pone.0051768.s001.doc]

**Supplementary table 1:** comparison between participants included and participants excluded because of missing baseline leptin or adiponectin data.

|  | **Excluded (N=406)** | **Included (n=3842)** | **Test (p-value)** |
| --- | --- | --- | --- |
| Women | 161 (39.7) | 2177 (56.7) | 42.92 (<0.001) |
| Age (years) | 50.5 ± 10.1 | 52.4 ± 10.5 | 3.40 (<0.001) |
| Body mass index (kg/m^2^) | 24.6 ± 5.2 | 25.4 ± 4.0 | 3.88 (<0.001) |
| Interleukin 1β (ng/l) | 1.23 (0.40 - 4.31) | 1.16 (0.48 - 3.81) | 0.01 (0.97) |
| Interleukin 6 (ng/l) | 1.56 (0.80 - 4.67) | 1.41 (0.69 - 3.29) | 5.18 (0.03) |
| Tumor necrosis factor-α (ng/l) | 2.75 (1.92 - 4.17) | 2.82 (1.78 - 4.43) | 0.01 (0.95) |
| hs-CRP (mg/l) | 0.9 (0.5 - 2.2) | 1.2 (0.6 - 2.6) | 8.82 (<0.005) |
| γGT (units/l) | 20 (14 - 29) | 20 (14 - 32) | 0.01 (0.92) |

CRP, C-reactive protein; γGT, gamma-glutamyl transpeptidase. Results are expressed as number of participants (percentage), as average ± standard deviation (age and BMI) or as median (interquartile range). Statistical analysis by chi-square or t-test.
