# Supplementary material for: Adipocytokines, Hepatic and Inflammatory Biomarkers and Incidence of Type 2 Diabetes. The CoLaus Study
Source: PLoS One. 2012 Dec 12;7(12):e51768. doi: 10.1371/journal.pone.0051768 (PMC3520903; doi:10.1371/journal.pone.0051768)
Supplement: Table S2 — Cytokine distribution between participants with (N = 208) and without (N = 3634) incident diabetes. (DOC) [file pone.0051768.s002.doc]

**Supplementary table 2**: cytokine distribution between participants with (N=208) and without (N=3634) incident diabetes

|  | **Non diabetic** | **Incident diabetes** | **P-value** |
| --- | --- | --- | --- |
| Interleukin 1β (ng/l) | 0.41 (0.10 – 1.77) | 0.30 (0.10 – 1.05) | 0.01 |
| Interleukin 6 (ng/l) | 1.22 (0.53 – 2.95) | 1.67 (0.73 – 3.65) | 0.003 |
| Tumour necrosis factor-α (ng/l) | 2.77 (1.70 – 4.39) | 3.00 (1.94 – 4.51) | 0.13 |
| hs-CRP (mg/l) | 1.1 (0.6 - 2.5) | 2.2 (1.2 - 4.1) | <0.001 |
| Leptin (µg/l) | 9.30 (5.05 - 16.77) | 11.76 (6.37 - 22.97) | <0.001 |
| Adiponectin (mg/l) | 8.72 (5.54 - 13.29) | 6.21 (4.07 - 9.06) | <0.001 |
| γGT (units/l) | 19 (14 - 30) | 34 (22 - 54) | <0.001 |

Results are expressed as median and (interquartile range) for values over detection level. Statistical analysis by Kruskall-Wallis nonparametric test
