# Supplementary material for: Adipocytokines, Hepatic and Inflammatory Biomarkers and Incidence of Type 2 Diabetes. The CoLaus Study
Source: PLoS One. 2012 Dec 12;7(12):e51768. doi: 10.1371/journal.pone.0051768 (PMC3520903; doi:10.1371/journal.pone.0051768)
Supplement: Table S3 — Association between adipocytokine, hepatic or inflammatory marker levels (as continuous log-transformed variables) and incident type 2 diabetes. (DOC) [file pone.0051768.s003.doc]

**Supplementary table 3**: Association between adipocytokine, hepatic or inflammatory marker levels (as continuous log-transformed variables) and incident type 2 diabetes.

|  | **No adjustment** | **Adjusted ^a^** | **Adjusted ^b^** | **Adjusted ^c^** |
| --- | --- | --- | --- | --- |
| IL-1β |  | 0.88 (0.72 - 1.07) | 0.92 (0.74 - 1.13) | 0.87 (0.74 - 1.04) |
| IL-6 | 1.11 (0.97 - 1.27) | 1.08 (0.93 - 1.24) | 1.03 (0.88 - 1.20) | 1.05 (0.88 - 1.24) |
| TNF-α | 1.08 (0.94 - 1.23) | 1.00 (0.87 - 1.16) | 0.95 (0.81 - 1.10) | 0.88 (0.74 - 1.05) |
| hs-CRP | 1.70 (1.48 - 1.95) | 1.69 (1.45 - 1.96) | 1.31 (1.11 - 1.55) | 1.12 (0.94 - 1.34) |
| Leptin | 1.39 (1.20 - 1.60) | 1.98 (1.66 - 2.35) | 1.19 (0.96 - 1.47) | 0.90 (0.77 - 1.06) |
| Adiponectin | 0.62 (0.54 - 0.71) | 0.65 (0.56 - 0.75) | 0.73 (0.62 - 0.86) | 0.84 (0.72 - 0.99) |
| γGT | 1.92 (1.71 - 2.16) | 1.68 (1.47 - 1.92) | 1.49 (1.29 - 1.72) | 1.26 (1.08 - 1.47) |

IL-1β, interleukin 1-β; IL-6, interleukin-6; TNF-α, tumour necrosis factor-α; CRP, C-reactive protein; γGT, gamma-glutamyl transpeptidase. Data from 208 participants who developed type 2 diabetes mellitus and 3634 controls. For IL-1β, IL-6 and TNF-α the sample size is smaller due to values below detection levels. Results are expressed as Odds ratio and (95% confidence interval) for an increase in one standard deviation of the log-transformed markers. Statistical analysis by logistic regression: ^a^, adjusting for age and gender; ^b^, adjusting for age, gender and body mass index; ^c^, adjusting for Kahn’s clinical and biological score.
