# Supplementary material for: Adipocytokines, Hepatic and Inflammatory Biomarkers and Incidence of Type 2 Diabetes. The CoLaus Study
Source: PLoS One. 2012 Dec 12;7(12):e51768. doi: 10.1371/journal.pone.0051768 (PMC3520903; doi:10.1371/journal.pone.0051768)
Supplement: Table S4 — Impact of adding different adipocytokine, hepatic or inflammatory markers (as continuous log-transformed variables) in the predictive capacity of a clinical + biological (C+B) risk score for type 2 diabetes. (DOC) [file pone.0051768.s004.doc]

**Supplementary table 4**: Impact of adding different adipocytokine, hepatic or inflammatory markers (as continuous log-transformed variables) in the predictive capacity of a clinical + biological (C+B) risk score for type 2 diabetes.

|  | **HL-test (p-value)** | **AIC** | **BIC** | **AROC § (95% CI)** | **AROC §§ (95% CI)** |
| --- | --- | --- | --- | --- | --- |
| Kahn’s C+B score | 0.84 | 1123.6 | 1136.1 | 0.901 (0.883 - 0.919) | 0.681 (0.648 - 0.715) |
| Kahn’s C+B score + IL-1β | 1.00 | 1123.2 | 1141.9 | 0.901 (0.883 - 0.919) | 0.692 (0.658 - 0.726) |
| Kahn’s C+B score + IL-6 | 1.00 | 1125.3 | 1144.1 | 0.901 (0.883 - 0.919) | 0.679 (0.645 - 0.712) |
| Kahn’s C+B score + TNF-α | 1.00 | 1123.6 | 1142.4 | 0.901 (0.882 - 0.919) | 0.687 (0.653 - 0.720) |
| Kahn’s C+B score + hs-CRP | 0.46 | 1124.0 | 1142.7 | 0.901 (0.883 - 0.919) | 0.686 (0.652 - 0.720) |
| Kahn’s C+B score + leptin | 1.00 | 1124.1 | 1142.8 | 0.901 (0.883 - 0.919) | 0.696 (0.662 - 0.730) |
| Kahn’s C+B score + adiponectin | 1.00 | 1121.2 | 1139.9 | 0.903 (0.886 - 0.920) | 0.684 (0.651 - 0.718) |
| Kahn’s C+B score + γGT | 1.00 | 1116.9 | 1135.7 | 0.903 (0.886 - 0.920) | 0.691 (0.657 - 0.725) |
| Kahn’s C+B score + all variables | 1.00 | 1117.9 | 1174.2 | 0.906 (0.890 - 0.922) | 0.705 (0.671 - 0.739) |

Statistical analysis by logistic regression. HL, Hosmer-Lemeshow goodness-of-fit test (only p-values are reported); AIC, Akaike’s information criterion; BIC, Bayesian information criterion; AROC, area under the ROC curve; IL-1β, interleukin 1 beta; IL-6, interleukin 6; TNF-α, tumour necrosis factor alpha; hs-CRP, high sensitive C reactive protein; γGT, gamma glutamyl transpeptidase. **§** using the type 2 diabetes risk predicted by the model as a continuous variable; **§§** splitting the type 2 diabetes risk into two categories (not at risk and at risk). Data from 208 participants who developed type 2 diabetes mellitus and 3634 controls. None of the models is significantly different (p<0.05) from the baseline model (Kahn’s C+B score).
