# Supplementary material for: Adipocytokines, Hepatic and Inflammatory Biomarkers and Incidence of Type 2 Diabetes. The CoLaus Study
Source: PLoS One. 2012 Dec 12;7(12):e51768. doi: 10.1371/journal.pone.0051768 (PMC3520903; doi:10.1371/journal.pone.0051768)
Supplement: Table S5 — Impact of adding different adipocytokine, hepatic or inflammatory markers as continuous log-transformed variables in the ability of a clinical + biological risk score to predict type 2 diabetes, using a 23% probability threshold to define high risk subjects. (DOC) [file pone.0051768.s005.doc]

**Supplementary table 5**: Impact of adding different adipocytokine, hepatic or inflammatory markers as continuous log-transformed variables in the ability of a clinical + biological risk score to predict type 2 diabetes, using a 23% probability threshold to define high risk subjects.

|  | **Sensitivity (%)** | **Specificity (%)** | **PPV (%)** | **NPV (%)** | **NRI (%)** | **IDI (%)** |
| --- | --- | --- | --- | --- | --- | --- |
| Kahn’s C+B score | 40.9 (34.1 - 47.9) | 95.4 (94.6 - 96.0) | 33.6 (27.8 - 39.8) | 96.6 (95.9 - 97.1) | - | - |
| Score + IL-1β | 42.8 (36.0 - 49.8) | 95.6 (94.9 - 96.3) | 35.9 (29.9 - 42.2) | 96.7 (96.1 - 97.2) | 2.17 (0.27; 4.07)* | 0.13 (-0.06; 0.46) |
| Score + IL-6 | 40.4 (33.7 - 47.4) | 95.4 (94.6 - 96.0) | 33.3 (27.5 - 39.5) | 96.5 (95.9 - 97.1) | -0.48 (-1.43; 0.46) | 0.01 (-0.07; 0.10) |
| Score + TNF-α | 41.8 (35.0 - 48.8) | 95.5 (94.8 - 96.1) | 34.7 (28.8 - 40.9) | 96.6 (96.0 - 97.2) | 1.07 (-0.83; 2.97) | 0.13 (-0.10; 0.36) |
| Score + hs-CRP | 41.8 (35.0 - 48.8) | 95.4 (94.6 – 96.0) | 34.1 (28.3 - 40.3) | 96.6 (96.0 - 97.2) | 0.96 (-1.36; 3.28) | 0.15 (-0.06; 0.36) |
| Score + leptin | 43.8 (36.9 - 50.8) | 95.4 (94.7 - 96.1) | 35.3 (29.4 - 41.4) | 96.7 (96.1 - 97.3) | 2.91 (0.60; 5.23)* | 0.00 (-0.21; 0.22) |
| Score + adiponectin | 41.3 (34.6 - 48.4) | 95.5 (94.8 - 96.2) | 34.7 (28.8 – 41.0) | 96.6 (96.0 - 97.2) | .145 (-2.19; 3.49) | 0.13 (-0.20; 0.46) |
| Score + γGT | 42.8 (36.0 - 49.8) | 95.4 (94.6 – 96.0) | 34.6 (28.8 - 40.8) | 96.7 (96.0 - 97.2) | 1.92 (-1.07; 4.92) | 0.42 (-0.14; 0.97) |

Results are expressed as percentage and (95% confidence interval). PPV, positive predictive value; NPV, negative predictive value; NRI, net reclassification improvement; IDI, integrated discrimination improvement; IL-1β, interleukin 1 beta; IL-6, interleukin 6; TNF-α, tumour necrosis factor alpha; hs-CRP, high sensitive C reactive protein; γGT, gamma glutamyl transpeptidase. Data from 208 participants who developed type 2 diabetes mellitus and 3634 controls. *, statistically significant at p<0.05.
