# Supplementary material for: Adipocytokines, Hepatic and Inflammatory Biomarkers and Incidence of Type 2 Diabetes. The CoLaus Study
Source: PLoS One. 2012 Dec 12;7(12):e51768. doi: 10.1371/journal.pone.0051768 (PMC3520903; doi:10.1371/journal.pone.0051768)
Supplement: Table S6 — Impact of adding specific combinations of adipocytokine, hepatic or inflammatory markers in the predictive capacity of a clinical + biological (C+B) risk score for type 2 diabetes, using a 23% probability threshold to define high risk subjects. (DOC) [file pone.0051768.s006.doc]

**Supplementary table 6**: Impact of adding specific combinations of adipocytokine, hepatic or inflammatory markers in the predictive capacity of a clinical + biological (C + B) risk score for type 2 diabetes, using a 23% probability threshold to define high risk subjects.

|  | **Sensitivity (%)** | **Specificity (%)** | **PPV (%)** | **NPV (%)** | **NRI (%)** | **IDI (%)** |
| --- | --- | --- | --- | --- | --- | --- |
| Kahn’s C+B score alone | 40.9 (34.1 - 47.9) | 95.4 (94.6 - 96.0) | 33.6 (27.8 - 39.8) | 96.6 (95.9 - 97.1) | - | - |
| Kahn’s C+B score +CRP+ adiponectin | 43.8 (36.9 - 50.8) | 95.6 (94.9 - 96.3) | 36.4 (30.4 - 42.7) | 96.7 (96.1 - 97.3) | 3.13 (-1.30, 7.57) | 1.48 (0.74,2.23)*** |
| Kahn’s C+B score + CRP + γGT | 40.9 (34.1 - 47.9) | 95.4 (94.7 - 96.1) | 33.9 (28.0 - 40.1) | 96.6 (95.9 - 97.1) | 1.37 (-2.63, 2.74) | 0.44 (-0.11, 0.99) |
| Kahn’s C+B score + all markers | 44.7 (37.8 - 51.7) | 95.4 (94.6 - 96.0) | 35.6 (29.8 - 41.8) | 96.8 (96.2 - 97.3) | 2.16 (-0.39, 8.09) | 2.09 (1.08,3.10)*** |

Results are expressed as percentage and (95% confidence interval). PPV, positive predictive value; NPV, negative predictive value; NRI, net reclassification improvement; IDI, integrated discrimination improvement; γGT, gamma glutamyl transpeptidase. Data from 208 participants who developed type 2 diabetes mellitus and 3634 controls. ***, p<0.001
